# Chemical Profiling, Analgesic and Anti-Inflammatory Activities of *Farsetia aegyptia* and *Zilla spinosa*: Integrated In Vitro, In Vivo, and In Silico Studies

**DOI:** 10.3390/plants15040523

**Published:** 2026-02-07

**Authors:** Malek Besbes, Assia Hamdi, Kaouther Majouli, Mabrouk Horchani, Abeer Ayed Alshammari, Saoussen Jilani, Salwa Ahmed Lotfi, Ramzi Hadj Lajimi, Hichem Ben Jannet, Walid Ben Selma, Jamil Kraiem

**Affiliations:** 1 Department of Biology, College of Sciences, University of Hail, Ha’il 81451, Saudi Arabia; a.ashammar@uoh.edu.sa (A.A.A.); s.jilani@uoh.edu.sa (S.J.); sal.abdelmohsen@uoh.edu.sa (S.A.L.); 2 Laboratory of Chemical, Pharmaceutical and Pharmacological Development of Drugs, Faculty of Pharmacy, University of Monastir, Monastir 5000, Tunisia; assia.hamdi@fphm.u-monastir.tn (A.H.); jamil.kraiem@fphm.u-monastir.tn (J.K.); 3 College of Science and Humanities–Al-Duwadmi, Shaqra University, Al-Duwadmi 11911, Saudi Arabia; kmajoli@su.edu.sa; 4 Laboratory of Heterocyclic Chemistry, Natural Products and Reactivity (LR11ES39), Faculty of Sciences of Monastir, University of Monastir, Monastir 5000, Tunisia; horchani.mabrouk@gmail.com (M.H.); hichem.benjannet@fsm.rnu.tn (H.B.J.); 5 Department of Chemistry, College of Science, University of Hail, Ha’il 81451, Saudi Arabia; r.lajimi@uoh.edu.sa; 6 Laboratory of Analysis, Treatment and Valorization of Environmental Pollutants and Products, Faculty of Pharmacy, University of Monastir, Monastir 5000, Tunisia; walid.bensalma@issatmh.u-monastir.tn

**Keywords:** Brassicaceae, anti-inflammatory, analgesic, anti-lipoxygenase, HR-LCMS, molecular docking

## Abstract

Plants are a rich source of active metabolites that have been used to treat inflammation troubles. The current study aimed to identify the analgesic and anti-inflammatory compounds in *Farsetia aegyptia* and *Zilla spinosa* extracts. The anti-inflammatory activity was evaluated using the xylene-induced ear edema model in mice and the carrageenan-induced paw edema model in Wistar rats. Additionally, both central and peripheral analgesic effects were assessed in mice. The anti-lipoxygenase activity was examined through an in vitro enzyme inhibition assay. The phytochemical composition of the bioactive extracts was characterized using High-Resolution Liquid Chromatography–Mass Spectrometry (HR-LCMS). The aqueous extracts of both species exhibited the strongest anti-inflammatory activity. The *F. aegyptia* extract showed inhibition percentages of 51.82% at 6.25 mg/kg and 51.14% at 0.78 mg/kg, while the *Z. spinosa* extract yielded 65.05% inhibition at 12.5 mg/kg and 56.14% at 1.56 mg/kg in the paw and ear edema models, respectively. These extracts also demonstrated significant analgesic activity and inhibited lipoxygenase, with IC_50_ values of 0.063 mg/mL for *F. aegyptia* and 0.072 mg/mL for *Z. spinosa*. HR-LCMS analysis revealed that the main constituent in Fa was malic acid (18.83%), while retronecine (19.03%) was the primary compound in *Z. spinosa*. Quercetin 3-[rhamnosyl-(1->2)-rhamnosyl-(1->6)-glucoside] was detected in both extracts with important proportions 7.87% in *F. aegyptia* and 8.29% in *Z. spinosa* and displayed the best docking score of −9.2 kcal/mol against the 5-lipoxygenase receptor (PDB: 3V99) in molecular docking studies. Overall, these findings indicate that *F. aegyptia* and *Z. spinosa* have significant potential as sources of novel anti-inflammatory agents.

## 1. Introduction

Inflammation is a complex immune response triggered by various factors, including infections, chemicals, and physical injuries. While it plays an essential role in tissue defense and repair, persistent or chronic inflammation can contribute to the onset and progression of numerous disorders, such as cardiovascular diseases, cancers, diabetes, and autoimmune conditions. Thus, understanding the mechanisms underlying inflammation is critical for developing effective therapeutic strategies [1,2]. Medicinal and aromatic plants serve as valuable sources of bioactive compounds, which can be broadly classified into primary and secondary metabolites. These phytochemicals hold significant promise for applications in food, cosmetics, and pharmaceuticals, with many still being investigated for their therapeutic potential [1]. Among these plants, the Brassicaceae family, also known as mustard, is particularly noteworthy. This family, distributed across North America, the Mediterranean, Asia, and Europe, comprises about 338 genera and 3709 species, many of which are cultivated worldwide for food, ornamental purposes, or industrial uses [3]. Brassicaceae species have long been integral to human diets, commonly consumed as fresh or preserved vegetables, condiments, and oils. They have also been widely used in traditional medicine from antiquity to the present [4,5]. This family has been traditionally associated with the management of inflammatory and pain-related conditions [6]. Epidemiological studies further associate diets rich in Brassicaceae with reduced systemic inflammation and a lower incidence of several cancers [7,8]. As a result, extracts and compounds derived from these plants are increasingly available in commercial markets [4]. Within the Brassicaceae family, *Farsetia aegyptia* Turra and *Zilla spinosa* (L.) Prantl are perennial shrubs that thrive in gravelly soils, sandy plains, stony wadis, and slopes. The selection of these species for the present study was supported by their ethnopharmacological relevance and phytochemical richness. *F. aegyptia* has long been utilized in folk medicine as an antirheumatic remedy and an antispasmodic, and for the therapy of inflammatory disorders and pain. In many arid countries, such as Kuwait and Morocco, decoctions of their aerial parts are traditionally prepared in water and used to relieve oral infections, gingivitis, sore eyes, and toothache, demonstrating a potent relationship with inflammatory conditions [9,10]. Phytochemical studies have shown that *F. aegyptia* contains phenolics, alkaloids, flavonoids, tannins, steroids, terpenes, saponins, and glycosides, as well as fatty acid derivatives such as linolenic and arachidonic acids, which are associated with several biological activities such as cytotoxic, allelopathic, antioxidant, antimicrobial, and inflammatory activities [9,10]. Likewise, *Z. spinosa* is commonly used in traditional medicine for gall bladder, kidney stones, gastrointestinal disorders, inflammatory conditions, and diabetes. Its chemical composition is defined by the presence of flavonoids, saponins, phenolic compounds, alkaloids, triterpenoids, and glycosides, with compounds such as β-sitosterol, stigmasterol, and lupeol being previously identified and reported for their anti-inflammatory, antimicrobial, and antioxidant properties [11,12,13]. Despite the ethnomedicinal overview, chemical profile, and biological activities, rigorous experimental evidence for the pharmacological activities, such as anti-inflammatory tests in both species, remains limited. This limitation motivated the present study to investigate their anti-inflammatory properties using in vivo and in vitro experimental models and to explore these species as essential sources of anti-inflammatory substances. The present study aims to compare the organic and aqueous extracts of *F. aegyptia* and *Z. spinosa*. Chloroform, ethanol, and water extracts from both species were tested for anti-inflammatory activity using xylene-induced ear edema and carrageenan-induced paw edema models. Analgesic activity was assessed through peripheral (acetic acid-induced writhing) and central (hot plate) methods. Additionally, anti-lipoxygenase (LOX) activity was evaluated using lipoxidase enzyme and linoleic acid as substrates. Bioactive extracts will be further characterized through high-resolution liquid chromatography–mass spectrometry (HR-LCMS) to correlate chemical profiles with biological effects. Finally, an in silico study will be conducted to integrate and enhance the biological and analytical findings.

## 2. Results

### 2.1. Acute Toxicity

The results indicated that among the extracts tested, *F. aegyptia* water extract was the most toxic sample, with an LD_50_ value of 263.91 mg/kg, followed by *Z. spinosa* water extract (LD_50_ = 249.04 mg/kg), *Z. spinosa* ethanolic extract (LD_50_ = 242.97 mg/kg), *F. aegyptia* ethanolic extract (LD_50_ = 222.03 mg/kg), *Z. spinosa* chloroformic extract (LD_50_ = 205.72 mg/kg), and *F. aegyptia* chloroformic extract (LD_50_ = 190.29 mg/kg). Based on these findings, we chose to test doses below the LD_50_.

### 2.2. Anti-Inflammatory Activity on Carrageenan-Induced Paw Edema in Rats and on Xylene-Induced Ear Edema in Mice

As shown in Table 1 and Table S1 (Supplementary File), paw edema in rats decreased over time. At a dose of 50 mg/kg, *F. aegyptia* ethanolic extract had the highest inhibition percentage (IP = 41.88 ± 0.54%) at the one-hour mark, compared to the reference drug diclofenac (IP = 40.30 ± 3.70%). The water extracts from both *F. aegyptia* water extract (IP = 37.71 ± 2.90%) and *Z. spinosa* water extract (IP = 37.08 ± 2.73%) also demonstrated significant anti-inflammatory effects. After three hours, *F. aegyptia* water extract emerged as the most effective with an IP of 71.16 ± 3.88%. At lower doses, after five hours, both *F. aegyptia* water extract (at 6.25 mg/kg, IP = 51.82 ± 1.60%) and *Z. spinosa* water extract (at 12.5 mg/kg, IP = 65.05 ± 0.17%) exhibited remarkable inhibition of edema.

The anti-inflammatory effects were further assessed in mice using xylene-induced ear edema. As presented in Table 2, all extracts at 50 and 25 mg/kg were effective against edema. The chloroformic extract from *F. aegyptia* was active at 6.25 mg/kg (58.48%) compared to that from *Z. spinosa*, for which the inhibition percentage was 44.24%. The ethanolic extracts from both species revealed statistically the same effect at 12.5 mg/kg (*F. aegyptia* ethanol 56.18%; *Z. spinosa* ethanol 56.36%). At lower concentrations than that of the standard, *F. aegyptia* water (at 0.78 mg/kg, IP = 51.14 ± 5.05%) and *Z. spinosa* water (at 1.56 mg/kg, IP = 56.14 ± 4.66%) reduced ear thickness more significantly than the reference drug dexamethasone (at 5 mg/kg, IP = 66.24 ± 3.10%). The same percentage inhibition was registered for dexamethasone (5 mg/kg, IP = 66.24%), *F. aegyptia* water (3.125 mg/kg; IP = 67.58%), and *Z. spinosa* water (3.125 mg/kg; IP = 62.50%) at different doses.

### 2.3. Analgesic Activity Using Acetic Acid and Hot-Plate Methods

The analgesic activity measured by two different methods is summarized in Table 3 and Table 4. All extracts at higher doses exhibited potent analgesic effects compared to the reference ASL in the acetic acid method. Notably, *F. aegyptia* water extract (0.78 mg/kg, IP = 52.45 ± 0.78%) and *Z. spinosa* water extract (1.56 mg/kg, IP = 53.31 ± 0.72%) were effective in reducing ear edema inflammation at lower doses. In the thermal method using a hot plate, both plant extracts showed analgesic activity after 30 min. However, these effects diminished over time at 60, 90, and 120 min, with *Z. spinosa* water extract (18.33 ± 0.58 s at 50 mg/kg) and *F. aegyptia* water extract (18.67 ± 0.58 s at 50 mg/kg) maintaining their activity even after 120 min. The lipoxygenase (LOX) inhibitory effect was evaluated by calculating the IC_50_ values (Table 5). The results showed that the *F. aegyptia* water extract (IC_50_ = 0.063 mg/mL) and *Z. spinosa* water extract (IC_50_ = 0.072 mg/mL) were the most effective against the lipoxygenase enzyme, followed by *F. aegyptia* chloroformic extract (IC_50_ = 0.108 mg/mL), *Z. spinosa* chloroformic extract (IC_50_ = 0.130 mg/mL), *F. aegyptia* ethanolic extract (IC_50_ = 0.114 mg/mL), and *Z. spinosa* ethanolic extract (IC_50_ = 0.146 mg/mL).

### 2.4. Analytical Analysis

The anti-inflammatory, analgesic, and anti-lipoxygenase tests demonstrated that the aqueous samples of the tested species produced the most favorable results by effectively reducing paw and ear edema, alleviating pain, and inhibiting lipoxygenase (LOX). Therefore, we selected these two samples for analysis via HR-LCMS. Table 6 summarizes their chemical composition, revealing 40 and 31 potential phytochemical compounds identified in the aqueous extracts of *F. aegyptia* and *Z. spinosa*, respectively. Notably, several of these phytocompounds are putatively detected for the first time in these plants on HR-LCMS evidence. The aqueous extract of *F. aegyptia* is particularly rich in compounds. The detected phytocompounds belong to various classes, including dipeptides, phenylpropanoids, alkaloids, flavonoids, carboxylic acids, phenolic compounds, polyols, lignans, coumarins, amino sugars, fatty acyls, amino alcohols, fatty acid amides, iridoids, and terpenoids. In the aqueous extract of *F. aegyptia*, the major compounds were the carboxylic acid; malic acid (area = 18.83%), the dipeptides; tyrosyl-leucine (area = 12.6%), isoleucyl-hydroxyproline (area = 7.71%), N-acetyl-leucyl-leucine (area = 5.81%), valyl-phenylalanine (area = 4.79%), the flavonoid glycoside quercetin 3-[rhamnosyl-(1→2)-rhamnosyl-(1→6)-glucoside] (area = 7.87%), kaempferol 3-(2G-apiosylrobinobioside) (area = 4.37%), CMP-N-glycoloylneuraminate (area = 4.40%). In contrast, the primary constituents in the aqueous extract from *Z. spinosa* include the alkaloids, retronecine (area = 19.03%), 2-(1,2,3,4-Tetrahydroxybutyl)-6-(2,3,4-trihydroxybutyl) pyrazine (4.11%), followed by the flavonoids glycoside quercetin 3-[rhamnosyl-(1→2)-rhamnosyl-(1→6)-glucoside] (area = 8.29%), quercetin 3-rhamnoside-7-glucoside (area = 6.05%), quercetin 3,7-dirhamnoside (area = 6.05%), and the dipeptide prolyl-arginine (area = 6.2%). Notably, some phytocompounds, such as gallic acid, malic acid, quercetin 3-[rham-nosyl-(1→2)-rhamnosyl-(1→6)-glucoside], quercetin 3,7-dirhamnoside, and CMP-N-glycoloylneuraminate, are common to both extracts.

### 2.5. In Silico Study

Molecular docking studies (MDS) were conducted using the 5-lipoxygenase target receptor (PDB: 3V99) to evaluate the molecular interactions associated with the experimental results obtained from the in vitro anti-lipoxygenase activity of *Z. spinosa* and *F. aegyptia* aqueous extracts. The binding site of eukaryotic lipoxygenases (LOXs) is known to be conserved, which led us to use LOX-5 as a structural model for determining the interactions formed. The binding site of LOX-5 consists primarily (approximately 50%) of hydrophobic amino acids, including Trp147, Phe169, Phe177, Leu179, Phe229, Leu368, Ile406, Ala410, Phe555, Ala603, Leu607, Phe610, Val671, and Ala672 [14]. This finding explains the enhanced activity of the ‘quercetin 3-[rhamnosyl-(1->2)-rhamnosyl-(1->6)-glucoside]’ which exhibited the best docking score of −9.2 kcal/mol compared to all other docked compounds, including the reference drug and the co-crystallized ligand. In comparison, the standard anti-inflammatory drug “Dexamethasone” achieved a docking score of −8.2 kcal/mol and exhibited some hydrophobic contacts with amino acids Phe177, Ile406, Ala410, and Leu607.

Table 7 summarizes that, among all the docked major compounds, besides to quercetin 3-[rhamnosyl-(1->2)-rhamnosyl-(1->6)-glucoside], two additional phytocompounds exhibited scores higher than that of the reference compound.

**Figure 1:**
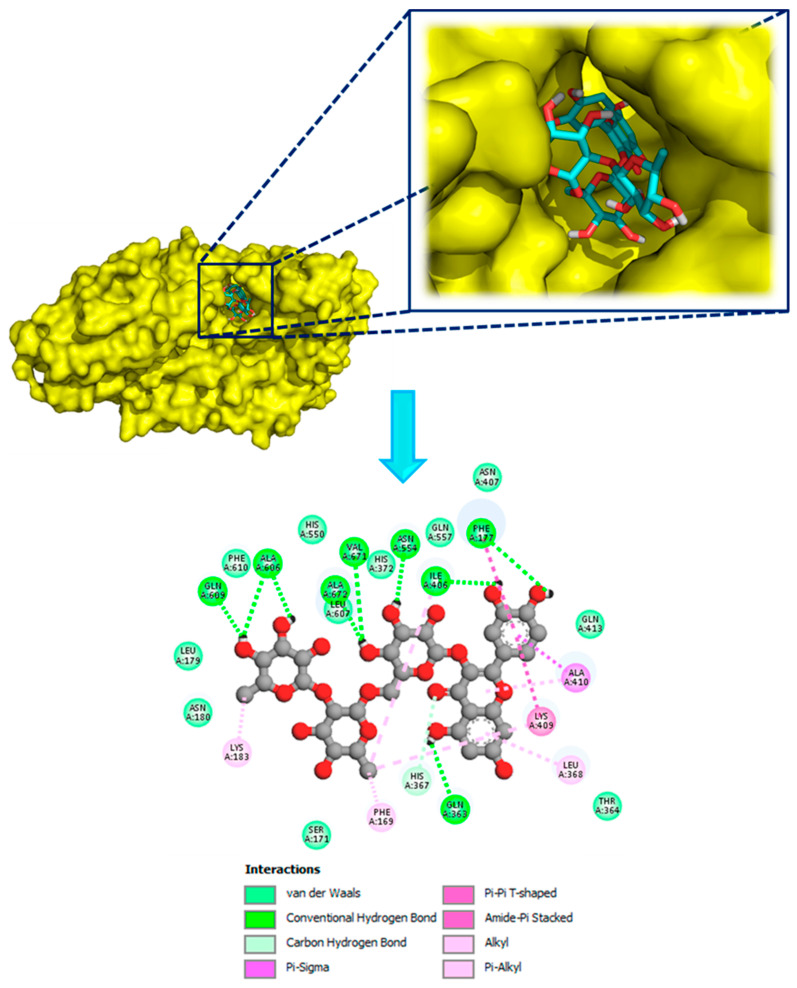
3D and 2D model of different interactions formed by the most active compound ‘Quercetin 3-[rhamnosyl-(1->2)-rhamnosyl-(1->6)-glucoside]’ within the active site of LOX (PDB: 3v99).

**Figure 2:**
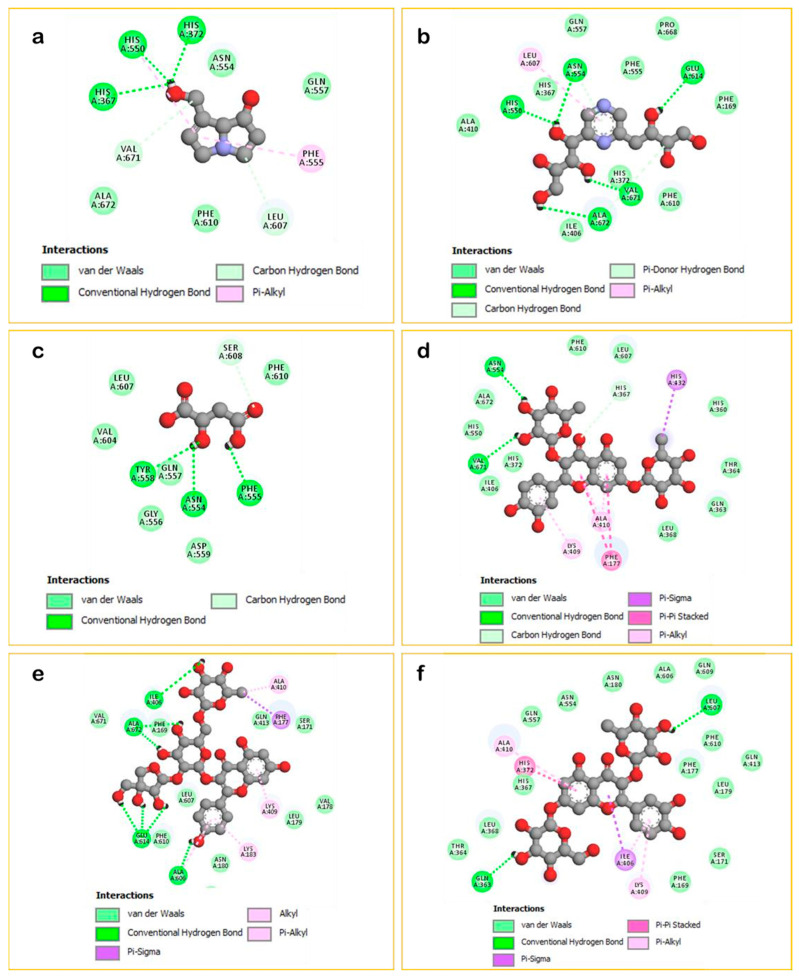
2D Binding modes of docked compounds: Retronecine (**a**), 2-(1,2,3,4-Tetrahydroxybutyl)-6-(2,3,4-trihydroxybutyl) pyrazine (**b**), Malic acid (**c**), Quercetin 3,7-dirhamnoside (**d**), Kaempferol 3-(2G-apiosylrobinobioside) (**e**), and Quercetin 3-rhamnoside-7-glucoside (**f**) within the binding site of LOX (PDB: 3v99).

**Figure 3:**
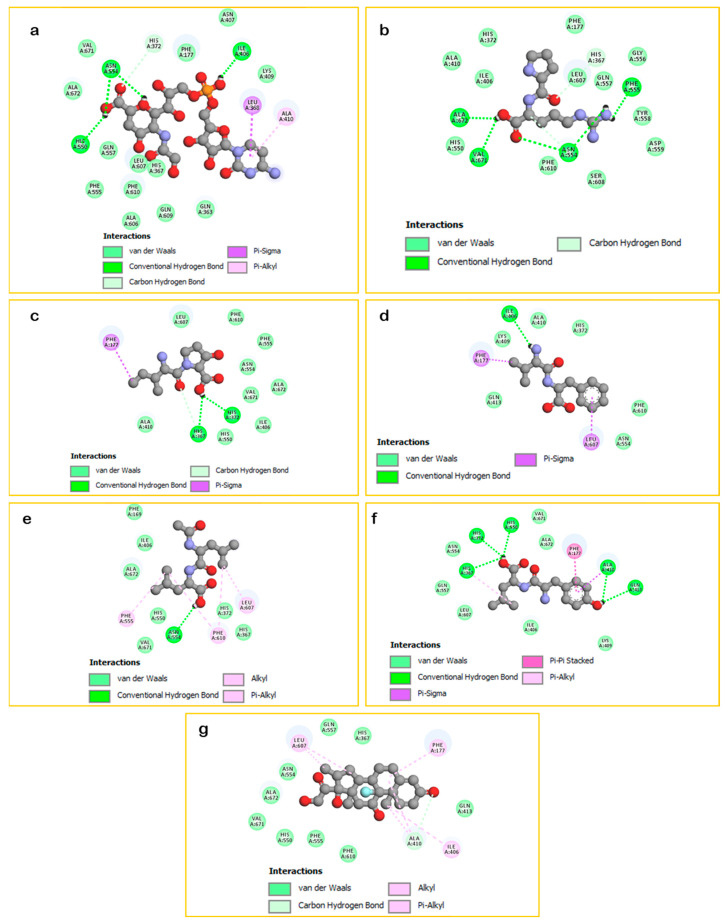
2D Binding modes of docked compounds: CMP-N-glycoloylneuraminate (**a**), Prolyl-arginine (**b**), Isoleucyl-Hydroxyproline (**c**), Valyl-Phenylalanine (**d**), N-Acetyl-leucyl-leucine (**e**), Tyrosyl-Leucine (**f**), and the standard: Dexamethasone (**g**) within the binding site of LOX (PDB: 3v99).

Figure 1 displayed that ‘quercetin 3-[rhamnosyl-(1→2)-rhamnosyl-(1→6)-glucoside]’ fits well in the binding cavity of the targeted enzyme. It formed several interactions, including nine hydrogen bonds with the amino acid sequence: Phe177, Gln363, Ile406, Asn554, Ala606, Gln609, Val671, and Ala672 in addition to other interactions as Carbon-hydrogen bond, Pi-Sigma, Pi-Pi T-shaped, Amide-Pi Stacked, Alkyls, and Pi-Alkyls. On another hand, as shown in Figure 2e, ‘quercetin 3,7-dirhamnoside’ (−8.7 kcal/mol) was found to be the second most active compound by exhibiting two H-Bonds with Asn554 and Val671 besides to some other contacts while the third most active ligand ‘kaempferol 3-(2G-apiosylrobinobioside)’ (−8.4 kcal/mol) showed seven H-Bonds with Ile406, Ala606, Glu614 and Ala672 in addition to some other interactions detailed in (Figure 2e). Moreover, all other docked phytoligands are involved in several interactions, especially the formation of H-Bonds (Figure 2 and Figure 3). Based on the outcomes of the in silico simulations, we can infer that the major phytocompounds derived from *Z. spinosa* and *F. aegyptia* extracts hold potential as noteworthy anti-inflammatory candidates.

## 3. Discussion

Traditional medicine across different cultures has long utilized multiple members of the *Brassicaceae* family to relieve inflammatory conditions and pain, largely through water-based preparations traditionally used in ethnobotanical remedies [15].

Both *Zilla spinosa* and *Farsetia aegyptia* have been employed to mitigate the aches and pains of rheumatic disorders, various inflammatory conditions, and to generally enhance a person’s wellbeing [9,10,11,12]. The study demonstrated that aqueous extracts displayed enhanced levels of anti-inflammatory, analgesic, and anti-lipoxygenase activity in comparison with both ethanolic and chloroformic extracts. Research indicates that the biological properties of these plant types are due to constituents that are soluble in water, such as the flavonoids and phenolic glycosides. The choice of aqueous extracts for further evaluation was based on the results of biological comparisons. This was underpinned by a similar traditional use and also by the comparative biological tests.

Acute toxicity testing is a common practice in the course of pre-clinical safety testing, aimed at establishing the LD_50_ values and safe experimental dosage ranges for toxicological studies in vivo. In the present research, no clinical signs of toxicity or mortality were observed up to the tested dose levels, showing the use of LD_50_ determination and behavioral observations to substantiate the selection of experimentally safe doses for further biological assessment. These methods are extensively adopted in preliminary in vivo pharmacological studies, ensuring a broad safety margin before efficacy evaluation [16]. The tested doses were selected as fractions of the LD_50_ to guarantee a wide safety margin, following widely accepted preclinical principles for plant extract work.

Literature shows that no previous studies have examined the in vivo toxicity of both *F. aegyptia* and *Z. spinosa*. However, these species are rich in various chemical compounds, which may partly explain their toxicity only at high doses. *Z. spinosa* contains a range of phytochemicals, including alkaloids, triterpenes, saponins, glycosides, progoitrin, goitrin, carbohydrates, sterols, and phenolic compounds like phenols and flavonoids [11,12,13,17]. Similarly, *F. aegyptia* contains alkaloids, alkenes, flavonoids, oximes, aldehydes, sugars, phenols, nitriles, fatty acids, steroids, ketones, esters, and amides [9,10,18,19,20]. Although both species are generally considered safe, the presence of these chemical classes, whether acting together or separately, can be toxic at high doses, potentially explaining the observed effects.

LOX inhibition was used in this study as a preliminary mechanistic indicator of the anti-inflammatory effect of the *F. aegyptia* and *Z. spinosa* extracts. The reported anti-inflammatory potential may be related to the modulation of multiple inflammatory pathways, as lipoxygenases comprise several isoforms with distinct pro or anti-inflammatory roles [21], consistent with the complex chemical profile of the tested samples. Additionally, to cyclooxygenase-related mechanisms, the lipoxygenase pathway and leukotriene-mediated responses can be involved in the reduction in edema and inflammatory signs shown in vivo. These multi-mechanistic activities may contribute to the observed efficacy and have been presented for secondary metabolites identified in Brassicaceae species [6], corroborating the pharmacological potential of the studied *F. aegyptia* and *Z. spinosa* extracts.

Past studies have shown that the chloroform extract from the aerial parts of *Z. spinosa* have anti-inflammatory effects in male rats, as demonstrated by its effect on carrageenan-induced paw edema. At 200 mg/kg, edema significantly decreased during the first three hours (28.68 ± 2.47% at 1 h, 34.31 ± 2.78% at 2 h, and 36.33 ± 2.42% at 3 h). In contrast, at 400 mg/kg, edema rates were 22.97 ± 2.75% at 1 h, 33.53 ± 1.94% at 2 h, and 29.86 ± 2.96% at 3 h [22]. These results are lower than ours, which showed that the chloroform extract inhibited edema by 51.07 ± 2.81% and 44.15 ± 1.81% at doses of 50 mg/kg and 25 mg/kg, respectively, after 3 h. Another study indicated that the chloroform fraction of the methanol extract reduced inflammation to 50% at 500 mg/kg and 44% at 250 mg/kg, which does not align with our observation of more potent effects at lower doses [23]. Hormetic dose–response effects, in which low concentrations of bioactive phytocompounds induce enhanced biological responses compared to higher doses, can be consistent with the high analgesic and anti-inflammatory activity seen at low doses [24]. In addition, by simultaneously altering many signaling pathways, the synergistic interactions between several compounds in the aqueous extracts may enhance their combined anti-inflammatory effects [25]. Furthermore, pro-inflammatory mediators and oxidative stress pathways are known to be modulated by plant phytochemicals to produce anti-inflammatory effects, offering a mechanistic explanation for the observed in vivo efficacy [26].

Additionally, the Bovine Serum Albumin (BSA) Protein Denaturation Assay was used to assess the anti-inflammatory activity of water, ethanolic, ethyl acetate, and butanolic extracts from *Z. spinosa*. Among these, the ethyl acetate, ethanolic, and butanolic extracts showed significant inhibition exceeding 50% at doses of 500 and 250 µg/mL. In contrast, the aqueous extract did not reach 50% inhibition at any tested dose (62.5, 125, 250, and 500 µg/mL) [27]. This difference may be due to variations between in vivo and in vitro methods. The anti-inflammatory effects seen in both *Z. spinosa* and *F. aegyptia* can be linked to their rich chemical makeup, including flavonoids, alkaloids, steroids, phenols, sterols, coumarins, saponins, and phytosterols [11,17,18], which are known for various pharmacological activities, especially anti-inflammatory properties [28]. For example, some isolated components from these species are already recognized for their anti-inflammatory effects [18,22,23], such as bergapten, psoralene, umbelliferone, β-amyrin, friedelene, campesterol, spinasterol, β-sitosterol, and stigmasterol from *Z. spinosa*’s chloroform extracts [17]. Furthermore, *F. aegyptia* contains components like amino acids, organic acids, glucosinolates, phenolic acids, C-glycosyl flavones, flavones, and flavonols, which may also help their anti-inflammatory effects [10]. Past studies reported that the chloroform extract of *Z. spinosa* reduced writhing by 74.48% (24.25 ± 1.41 writhes/20 min) at 200 mg/kg and by 77.08% (22.40 ± 0.45 writhes/20 min) at 400 mg/kg [17]. However, our work found that the chloroform extract had strong analgesic effects at lower doses, decreasing writhing by 55.79 ± 1.43%, 73.97 ± 2.48%, and 77.27 ± 3.79% at 12.5 mg/kg, 25 mg/kg, and 50 mg/kg, respectively. Both *Z. spinosa* and *F. aegyptia* contain many chemicals that may provide analgesic effects [17,18]. So far, no studies have specifically looked at the anti-lipoxygenase activity of *Z. spinosa* and *F. aegyptia* extracts. Yet, the presence of steroids, sterols, and phytosterols, like 26-Homo-25-Hydroxy cholesterol, sterol-β-D-glucopyranoside, campesterol, spinasterol, β-sitosterol, and stigmasterol, probably helps inhibit lipoxygenase enzymes [17,18]. Past research confirmed that these species are rich in flavonoids, phenols, alkaloids, and coumarins [13,17,19,20]. For example, kaempferol, quercetin derivatives, isorhamnetin derivatives, luteolin C-glycoside derivatives, phenolic acids, glucosinolates, and gallic acid have been found in *F. aegyptia* from Egypt [10,20]. Similarly, quercetrin was detected in *Z. spinosa*’s aqueous ethanol extract [11], and quercetin derivatives like quercetin 3-rhamnoside-7-glucoside and quercetin 3,7-dirhamnoside were found in *Z. spinosa* from Qassim, Saudi Arabia [29]. These earlier studies align with our findings, as these phytochemicals likely contribute to the notable activity in both species. Alkaloids have shown strong anti-inflammatory activity in xylene-induced ear swelling tests in mice [30]. Other research indicates that some peptides and amino acids can significantly reduce inflammation by decreasing levels of inducible nitric oxide synthase and inhibiting transcription factors such as NF-κB and STAT, along with markers like TNF-α, IL-1, IL-6, IL-8, and CRP [31,32]. Interestingly, one study identified tryptophan, an amino acid, in *F. aegyptia*’s methanol-water extract [10]. In our study, six dipeptides were putatively annotated for the first time based on HR-LCMS. Flavonoid glycosides are prevalent in our samples, making up 23.58% in *Z. spinosa* and 20.16% in *F. aegyptia*, and they showed strong binding energies against 5-lipoxygenase (PDB:3V99), with quercetin 3-[rhamnosyl-(1→2)-rhamnosyl-(1→6)-glucoside] demonstrating the best affinity (−9.2 kcal/mol). It was followed by quercetin 3,7-dirhamnoside (−8.7 kcal/mol), kaempferol 3-(2G-apiosylrobinobioside) (−8.4 kcal/mol), and quercetin 3-rhamnoside-7-glucoside (−8 kcal/mol). These results are comparable to or better than dexamethasone (reference ligand) at −8.2 kcal/mol. As is known, forming hydrogen bonds with residues in the receptor’s active site plays a vital role in inhibiting the target enzyme. In fact, the analysis of the tested molecules, particularly the three phytocompounds with more favorable docking scores than the reference drug, shows that these phytoligands interact with the target through a significant number of hydrogen bonds. Conversely, the standard compound dexamethasone did not form any H-bonds and engaged only in hydrophobic interactions. This suggests that their complex glycosylation structures may enhance stability within the 5-LOX active site. The in silico analysis showed a stable binding mode where kaempferol/quercetin aglycones bind within the hydrophobic catalytic channel, while the sugar chains interact with polar residues at the cavity entrance, forming additional hydrogen bonds that increase binding affinity. This mode aligns with recent findings showing that related flavonoid glycosides and phenolic O-glycosides like emodin-8-O-glucoside, scutellarin, and baicalin are effective at inhibiting 5-LOX [33]. Overall, these results support the idea that glycosides based on kaempferol and quercetin are major contributors to the predicted anti-inflammatory effects of the extracts through efficient targeting of 5-LOX.

Water extracts of *Z. spinosa* and *F. aegyptia* showed the strongest anti-inflammatory and pain-relief activities, exceeding those of ethanolic and chloroform extracts. High-resolution liquid chromatography–mass spectrometry (HR-LCMS) identified retronecine (19.03%) and quercetin 3-[rhamnosyl-(1→2)-rhamnosyl-(1→6)-glucoside] (8.29%) as the most common compounds in *Z. spinosa*, while malic acid (18.83%) and Tyrosyl-Leucine (12.6%) were predominant in *F. aegyptia*. These main compounds, along with minor metabolites, likely work together or add up to produce the observed effects. Malic acid has been shown to influence inflammatory pathways, including M1 macrophage polarization, supporting its role in the anti-inflammatory properties of *F. aegyptia* [34]. The pyrrolizidine alkaloid retronecine, found in *Z. spinosa*, might impact biological activity; its direct anti-inflammatory effects need more research [35]. Flavonoid glycosides, making up over 20% of the total, are known for their ability to reduce inflammation and probably play a key role in both species’ pharmacological effects [36].

The current investigation was developed as initial pharmacological research to validate the anti-inflammatory activity of traditionally used Brassicaceae species. Further studies examining compound fractionation and targeted mechanistic analyses can complement these results and supply deeper insight into the molecular pathways underlying.

## 4. Materials and Methods

### 4.1. Reagents and Chemicals

All solvents used in this study—ethanol (99.99%, C_2_H_6_O) and chloroform (90%, CHCl_3_)—were purchased from Merck (Darmstadt, Germany). Lipoxygenase (LOX) enzyme was obtained from Sigma-Aldrich (St. Louis, MO, USA; catalog no. L7395-15MU). The enzyme is lipoxygenase derived from *Glycine max* (soybean). The product was supplied for research use only and stored at 2–8 °C in accordance with the manufacturer’s recommendations. Diclofenac, lysine acetylsalicylate, and dexamethasone were provided by Adwya Laboratory (Tunis, Tunisia).

### 4.2. Plant Material and Extraction

*F. aegyptia* and *Z. spinosa* materials (1.6 kg) were collected from farms in the Simira governorate, located south of Hail, in February 2022, with verbal permission from the owners. The plants were taxonomically identified at the Faculty of Pharmacy, University of Monastir, Tunisia. They were confirmed to be *Farsetia aegyptia* and *Zilla spinosa*, both belonging to the family Brassicaceae, through macroscopic and microscopic examination.

A voucher specimen (F.a 1 and Z.s 1) was potted in the herbarium of the laboratory of biology (HBL) in the College of Sciences (Simira branch, Hail University). The plants were air-dried at normal room temperature and well-ventilated for a period of two weeks. The aerial parts of plants were broken into pieces before subjected into mechanical grinding, and transformed into a powdery form. Each plant powder (700 g) was successively extracted with chloroform (4 × 500 mL), ethanol (4 × 500 mL), and water (4 × 500 mL) at room temperature for 72 h. Thereafter, the resultant solutions were filtered through Whatman filter paper No. 1 grade. The chloroform and ethanol extracts were evaporated and concentrated at 60 °C under reduced pressure, using a rotary evaporator; the aqueous extracts were dried by lyophilization. The chloroform, ethanolic, and aqueous extracts were kept at −4 °C until utilization in sterile glass bottles [37].

### 4.3. Animals

Adult Wistar rats weighing between 170 and 200 g were prepared for the anti-inflammatory activity testing. Swiss albino mice weighing 18–30 g were used to evaluate acute toxicity and analgesic effects. The animals were sourced from the Faculty of Pharmacy at the University of Monastir and the Pasteur Institute in Tunis, Tunisia. They were provided with a standard pellet diet and had access to water ad libitum. The Wistar rats were fasted overnight while having free access to water before the bioassays. The housing conditions and in vivo experiments were conducted according to the guidelines established by the European Union on Animal Care (Communautés Économiques Européennes (CEE) Council [86/609]).

### 4.4. Acute Toxicity

To evaluate acute toxicity, different doses of 300, 200, 100, and 50 mg/kg were administered intraperitoneally (i.p.). The control group received 0.9% NaCl at a dosage of 10 mL/kg. The lethal doses (LD50) were determined by monitoring mouse mortality over 24 and 48 h [38]. After establishing the mortality rate, pharmacological doses were tested [39].

### 4.5. Anti-Inflammatory Assay on Carrageenan-Induced Paw Edema

This test was conducted according to the protocols established by Hamdi et al. [39] and Winter et al. [40]. The control group received an intraperitoneal (i.p.) dose of saline solution (NaCl 0.9%, 2.5 mL/kg). The reference group was administered i.p. diclofenac (25 mg/kg) from Adwya Laboratory in Tunis, Tunisia. The test groups received the samples i.p. at different doses. After 30 min, 50 µL of a 1% carrageenan suspension was injected into the left hind paw. The paw volume, measured up to the tibiotarsal articulation, was recorded using a plethysmometer (model 7150, Ugo Basile, Italy). Measurements were taken at 0 h (V0) before carrageenan injection and then at 1, 2, 3, 4, and 5 h (Vt) after the injection. The paw swelling for each rat was determined, and the difference between Vt and V0 was used to calculate the edema value. The percentage of inhibition was calculated using the following formula:

% inhibition = [(V_t_ − V_0_)_control_ − (V_t_ − V_0_)_treated group_]/(V_t_ − V_0_)_control_ × 100

### 4.6. Analgesic Assay

#### 4.6.1. Acetic Acid Method

The peripheral antinociceptive assay was conducted to investigate the effects of certain extracts [39,41]. This was assessed using the acetic acid abdominal constriction test, also known as the writhing test, which is a chemical model for visceral pain. Swiss albino mice were selected one day before testing and divided into groups of six mice each. One group served as the control and was pretreated subcutaneously (s.c.) with 10 mL/kg of 0.9% saline. Another group received the reference drug, acetylsalicylate of lysine (ASL), at a dosage of 200 mg/kg via the same route. The remaining groups were administered intraperitoneally (i.p.) with 10 mL/kg of a 1% acetic acid solution 30 min after receiving the extracts at different doses. The number of writhes was counted for 30 min following acetic acid administration. Antinociceptive activity was expressed as the percentage inhibition of the typical number of writhes observed in the control group, calculated using the following formula:

% Inhibition = [(number of writhes in control) − (number of writhes in treated group)]/(number of writhes in control) × 100

#### 4.6.2. Hot-Plate Test

The hot-plate method was employed to determine the central analgesic effect of the samples. Central antinociceptive activities against thermal noxious stimuli were evaluated in Swiss albino mice [39,42]. Mice were individually placed on a hot-plate analgesimeter (Ugo Basile, No. 35100, Varese, Italy), maintained at a temperature of 55–60.5 °C. The latency period, defined as the time between placement on the hot plate and either licking of the paws or jumping, was recorded in seconds. To reduce variability among the animals, those with latency times exceeding 15 s during pre-testing were excluded from the experiment [43]. Mice were grouped into sets of six. The control group was pretreated subcutaneously with 10 mL/kg of 0.9% saline, while the standard group received the reference drug ASL at 200 mg/kg (s.c.). The remaining groups were injected with various doses of extracts: *F. aegyptia* chloroformic (12.5, 25, and 50 mg/kg), *F. aegyptia* ethanolic (25 and 50 mg/kg), *F. aegyptia* water (6.25, 12.5, 25, and 50 mg/kg), *Z. spinosa* chloroformic (25 and 50 mg/kg), *Z. spinosa* ethanolic (25 and 50 mg/kg), and *Z. spinosa* water (6.25, 12.5, 25, and 50 mg/kg) subcutaneously. The latency time was recorded at 30, 60, 90, and 120 min after s.c. administration.

### 4.7. Anti-Lipoxygenase Activity

Anti-lipoxygenase activity can be assessed using lipoxygenase enzymes. In the present study, 15-lipoxygenase (15-LOX) was used as a preliminary screening enzyme to evaluate the lipoxygenase inhibitory potential of the plant extracts. To study this activity, the lipoxygenase enzyme is incubated under specific conditions with inhibitors (e.g., herbal extracts) in the presence of linoleic acid as the substrate [44,45]. Various concentrations (5–0.039 mg/mL) of chloroformic, ethanolic, and aqueous extracts from both species were dissolved in 0.25 mL of 2 M borate buffer (pH 9.0). The mixture was then combined with 0.25 mL of soybean lipoxygenase enzyme solution. After 5 min of incubation at 25 °C, 1 mL of linoleic acid solution was added, and the absorbance was measured at 234 nm. Dexamethasone (60 µg/mL) was used as a general reference anti-inflammatory drug for comparative purposes in the discussion of plant extract activity. The following equation was applied to calculate the percentage inhibition: % IP = ([Absorbance of control − Absorbance of test sample]/Absorbance of control) × 100. The IC_50_ values of the tested samples were also determined.

### 4.8. Phytochemical Profile of Farsetia aegyptia and Zilla spinosa Aqueous Extracts

The phytochemical analysis of *F. aegyptia* and *Z. spinosa* aqueous extracts was performed using a UHPLC-PDA-High-Resolution Mass Spectrometer (1290 Infinity UHPLC system) from Agilent Technologies^®^, Santa Clara, CA, USA. The liquid chromatographic system included a HiP sampler, a binary gradient solvent pump, a column compartment, and a quadrupole time-of-flight mass spectrometer (MS Q-TOF) equipped with a dual Agilent Jet Stream Electrospray (AJS ES) ion source (Agilent Technologies, Santa Clara, CA, USA). A sample volume of 3 µL was injected into the system and separated on an SB-C18 column (2.1 mm × 50 mm, 1.8 µm particle size) maintained at 40 °C. Solvent A consisted of 0.1% formic acid in deionized water, while solvent B was acetonitrile, with a flow rate set at 0.300 mL min^−1^. Mass spectrometric detection was performed in positive-ion MS Q-TOF mode using AutoMS^2^ acquisition over *m*/*z* 120–1200. Putative compound annotation was based on accurate mass measurements, isotopic pattern agreement, and MS/MS fragmentation patterns acquired with medium isolation width (~4 amu) and ramped collision energy. Accordingly, all HR-LCMS compound assignments are reported as putatively annotated metabolites corresponding to Metabolomics Standards Initiative (MSI) Level 2. The main tools used for phytochemical annotation included ChemSpider and PubChem databases [46]. The mass spectrometer was routinely calibrated before analysis, and analyses were carried out under identical conditions to ensure repeatability.

### 4.9. In Silico Study

Molecular docking studies were performed using the AutoDock 4.2 program package [47]. The X-ray crystallographic structure of Stable-5-LOX in complex with Arachidonic Acid (PDB: 3v99) was obtained from the RCSB Protein Data Bank 1 [48]. The geometries of all compounds were optimized using ACD (3D Viewer (2017 version)) software. For receptor preparation, water molecules (H_2_O) were removed, missing hydrogens were added, and Gasteiger charges were incorporated into the system during the preparation of the receptor input file (PDBQT). The visualization and analysis of interactions were conducted using Discovery Studio 2017R2.

### 4.10. Statistical Analysis

GraphPad Prism version 9 and Microsoft Excel 2019 were used to analyze the biological tests. Mean ± standard deviation (SD) and, when applicable, percentages are used to express the data. Data were examined for homogeneity of variances and normality before analysis.

One-way analysis of variance (ANOVA) was used to evaluate treatment differences. Duncan’s multiple range test served as a post hoc method to compare means when significant differences (*p* < 0.05) were detected.

By fitting the data to a four-parameter logistic model, nonlinear regression analysis of the concentration–response curves in GraphPad Prism was used to calculate the IC_50_ values for enzyme inhibition. This process enabled the determination of the concentration at which enzyme activity was inhibited by 50%. SPSS version 16.0 was used for all statistical analyses.

## 5. Conclusions

This work reports experimental evidence supporting the anti-inflammatory, analgesic, and anti-lipoxygenase activities of *F. aegyptia* and *Z. spinosa*, particularly in their water samples, in line with their traditional use. HR-LCMS profiling demonstrated a chemically diverse composition, with peptides, organic acids, flavonoid glycosides, and alkaloids among the predominant phytocompounds. Whereas constituents such as malic acid, retronecine, and flavonoid glycosides were identified at relatively high abundance, the recorded biological effects are likely the result of the combined action of multiple phytocompounds instead of a single compound.

Importantly, the present study should be viewed with the limitations of crude extracts and predictive computational studies, which do not allow for a definitive assignment of activity to specific metabolites. Therefore, future research involving compound isolation, fractionation, and targeted mechanistic studies is necessary to identify the precise contributors and pathways behind the observed effects. Overall, these findings support the pharmacological relevance of both species and lay the groundwork for future, more detailed mechanistic investigations.

## Figures and Tables

**Table 1:** Anti-inflammatory effect of *Zilla spinosa* and *Farsetia aegyptia* extracts on carrageenan-induced paw edema in rats.

Samples	Percentage Inhibition of Paw Edema (%)			
	Dose mg/Kg	1 h	3 h	5 h
Vehicle (mm)		3.32	4.65	3.43
Fac	50	27.29 f	54.82 de	70.81 e
	25	22.71 e	50.85 d	62.12 d
	12.5	20.63 e	43.49 bc	44.14 ab
Fae	50	41.88 i	67.33 g	72.22 ef
	25	14.79 d	47.46 c	62.42 d
	12.5	4.69 a	39.88 b	45.96 b
Faw	50	37.71 h	71.16 h	79.49 f
	25	36.56 h	61.22 f	70.91 e
	12.5	31.77 fg	62.47 f	70.91 e
	6.25	13.13 d	41.50 b	51.82 d
	3.125	8.02 c	32.16 a	42.53 a
Zsc	50	14.58 d	51.07 d	70.30 e
	25	1438 d	44.15 bc	51.21 d
	12.5	7.81 bc	31.05 a	40.00 a
Zse	50	34.79 gh	66.89 g	80.61 g
	25	30.00 fg	51.29 d	69.49 e
	12.5	6.25 b	41.80 b	46.06 c
Zsw	50	37.08 h	58.06 e	63.43 d
	25	36.77 h	57.10 e	68.59 e
	12.5	34.17 gh	55.26 de	65.05 de
	6.25	27.71 f	41.06 b	48.99 c
Diclofenac	25	40.30 i	68.91 g	74.76 ef

Fac: *Farsetia aegyptia* chloroformic extract, Fae: *Farsetia aegyptia* ethanolic extract, Faw: *Farsetia aegyptia* water extract, Zsc: *Zilla spinosa.* chloroformic extract, Zse: *Zilla spinosa* ethanolic extract, Zsw: *Zilla spinosa* water extract. Diclofenac was used as a reference anti-inflammatory drug. The letters (a–i) indicate a significant difference among the extract doses according to the Duncan assay (*p* < 0.05).

**Table 2:** Inhibitory effect (%) of *Zilla spinosa* and *Farsetia aegyptia* samples on xylene-induced ear edema in mice.

Dose mg/Kg	Samples						Control	Reference
	Fac	Fae	Faw	Zsc	Zse	Zsw	Vehicle	Dex (5 mg/mL)
							0.11	66.24
50	80.91 a	77.95 a	97.05 c	82.73 ab	80.00 a	96.67 c		
25	78.18 b	64.73 a	88.18 c	75.27 b	60.18 a	94.55 d		
12.5	57.50 a	56.18 a	83.64 c	65.45 b	56.36 a	85.68 c		
6.25	58.48 d	36.36 b	70.23 e	44.24 c	21.82 a	77.88 f		
3.125	36.06 a		67.58 c			62.50 b		
1.56			58.41			56.14		
0.78			51.14			34.55		
0.39			32.42					

Fac: *Farsetia aegyptia* chloroformic extract, Fae: *Farsetia aegyptia* ethanolic extract, Faw: *Farsetia aegyptia* water extract, Zsc: *Zilla spinosa* chloroformic extract, Zse: *Zilla spinosa* ethanolic extract, Zsw: *Zilla spinosa* water extract, Dex: dexamethasone. The letters (a–f) show an important difference between the different doses of extracts in accordance with the Duncan assay (*p* < 0.05).

**Table 3:** Analgesic activity of *Zilla spinosa* and *Farsetia aegyptia* extracts evaluated by the acetic acid-induced writhing test in mice.

% Inhibition of Writhing (Acetic Acid)								
Samples (mg/kg)	50	25	12.5	6.25	3.125	1.562	0.78	0.39
Zsc	77.27 a	73.97 bc	55.79 a	36.36 b				
Zse	79.75 a	62.40 a	57.85 a	50.83 c	25.62 a			
Zsw	83.47 b	78.93 c	75.21 c	72.31 e	68.18 d	53.31	42.15	
Fac	76.67 a	70.39 b	63.21 b	31.81 a				
Fae	78.02 a	68.60 b	60.07 b	56.03 d	40.78 b			
Faw	81.61 ab	80.71 d	77.57 c	75.33 ef	64.56 c	57.83	52.45	10.72
ASL 200 mg/Kg	66.53							
Vehicle (writhes)	80.67							

Fac: *Farsetia aegyptia* chloroformic extract, Fae: *Farsetia aegyptia* ethanolic extract, Faw: *Farsetia aegyptia* water extract, Zsc: *Zilla spinosa* chloroformic extract, Zse: *Zilla spinosa* ethanolic extract, Zsw: *Zilla spinosa* water extract. ASL: acetylsalicylate of lysine. Different letters (a–f) within the same column show an important difference between the different doses of extracts in accordance with the Duncan assay (*p* < 0.05).

**Table 4:** Analgesic activity of *Zilla spinosa* and *Farsetia aegyptia* extracts evaluated by the hot plate test in mice at different time intervals.

Times (min)					
Samples	Dose (mg/kg)	30	60	90	120
Vehicle		8.33 a	9.67 a	7.67 a	5.33 ab
ASL	200	25.33 d	23.33 d	20.00 d	16.33 cd
Zsc	50	23.00 c	20.00 d	14.67 c	9.33 b
	25	18.00 bc	12.33 b	10.00 b	5.33 ab
Zse	50	23.00 c	1.00 c	15.67 c	8.33 b
	25	18.33 bc	17.67 c	10.67 b	4.33 a
Zsw	50	25.00 d	23.33 d	20.00 d	18.33 d
	25	23.67 c	20.33 d	18.33 cd	13.67 c
	12.5	17.67 bc	14.33 b	10.33 b	7.67 b
	6.25	15.00 b	11.33 b	8.67 a	4.33 a
Fac	50	22.67 c	20.33 d	13.33 bc	8.67 b
	25	20.33 c	17.67 c	13.33 bc	6.33 ab
	12.5	14.00 b	8.33 a	5.33 a	3.00 a
Fae	50	23.33 c	18.33 c	15.00 c	8.67 b
	25	15.33 b	10.33 ab	7.67 a	3.67 a
Faw	50	25.67 d	23.33 d	19.67 d	18.67 d
	25	24.33 cd	21.67 d	19.00 d	13.00 c
	12.5	18.67 bc	14.33 b	10.67 b	7.00 b
	6.25	14.33 b	8.00 a	5.67 a	3.33 a

Fac: *Farsetia aegyptia* chloroformic extract, Fae: *Farsetia aegyptia* ethanolic extract, Faw: *Farsetia aegyptia* water extract, Zsc: *Zilla spinosa* chloroformic extract, Zse: *Zilla spinosa* ethanolic extract, Zsw: *Zilla spinosa* water extract. ASL: acetylsalicylate of lysine. Different letters (a–d) within the same column show an important difference between the different doses of extracts in accordance with the Duncan assay (*p* < 0.05).

**Table 5:** Anti-lipoxygenase activity of *Farsetia aegyptia* and *Zilla spinosa* extracts (IC_50_, mg/mL).

	Samples						
	Zsc	Zse	Zsw	Fac	Fae	Faw	Dex
IC_50_ (mg/mL)	0.130 ± 0.001 f	0.146 ± 0.003 g	0.072 ± 0.003 c	0.108 ± 0.003 d	0.114 ± 0.002 e	0.063 ± 0.007 b	0.042 ± 0.002 a

Fac: *Farsetia aegyptia* chloroformic extract, Fae: *Farsetia aegyptia* ethanolic extract, Faw: *Farsetia aegyptia* water extract, Zsc: *Zilla spinosa* chloroformic extract, Zse: *Zilla spinosa* ethanolic extract, Zsw: *Zilla spinosa* water extract. Dex: Dexamethasone (positive control). Different letters (a–g) indicate statistically significant differences among samples according to Duncan’s multiple range test (*p* < 0.05).

**Table 6:** Chemical composition of *Farsetia aegyptia* and *Zilla spinosa* aqueous extracts identified by HR-LCMS.

Compounds	Class of Compound	RT (min)	Formula	[M^+^H]^+^ *(m*/*z)*	[M^−^H]^−^ *(m*/*z)*	Area (%) *F. aegyptia*	Area (%) *Z. spinosa*
Retronecine	Alkaloid	1.48	C_8_H_13_NO_2_	156.1013	-	-	19.03
Quinic acid	Polyol	1.51	C_7_H_12_O_6_	-	191.0556	-	1.52
2-(1,2,3,4-Tetrahydroxybutyl)-6-(2,3,4-trihydroxybutyl) pyrazine	Alkaloid	1.81	C_12_H_20_N_2_O_7_	305.1321	-	-	4.11
1-O-2′-Hydroxy-4′-methoxycinnamoyl-b-D-glucose	Phenylpropanoids	1.87	C_16_H_20_O_9_	-	355.1004	0.63	-
Acrimarine J	Benzoquinolines	1.92	C_35_H_33_NO_8_	-	594.2161	-	0.83
Citric acid	Carboxylic acids	1.96	C_6_H_8_O_7_	-	191.0196	-	1.92
10-Acetoxyligustroside	Terpene glycosides	1.97	C_27_H_34_O_14_	-	581.1843	-	1.4
Prolyl-Arginine	Dipeptide	2.155	C_11_H_21_N_5_O_3_	294.1521	-	-	6.2
N-Ethylglycocyamine	Polyol	2.16	C_8_H_17_NO_5_	230.1006	-	-	1.42
Gallic acid	Phenylpropanoids	2.24	C_7_H_6_O_5_	-	169.015	0.06	0.76
Malic acid	Carboxylic acids	2.46	C_4_H_6_O_5_	-	133.0143	18.83	3.81
Isoleucyl-Hydroxyproline	Dipeptide	2.32	C_11_H_20_N_2_O_4_	267.131	-	7.71	-
Valyl-Phenylalanine	Dipeptide	2.967	C_14_H_20_N_2_O_3_	265.1518	-	4.79	-
L-isoleucyl-L-proline	Dipeptide	3.435	C_11_H_20_N_2_O_3_	229.152	-	1.80	-
Gentisic acid	Phenolic acids.	3.47	C_7_H_6_O_4_	-	153.0206	0.1	-
(Z)-2-Methyl-2-butene-1,4-diol 4-O-beta-D-Glucopyranoside	Glycoside	3.53	C_11_H_20_O_7_	-	323.1352	0.58	-
N-Acetyl-leucyl-leucine	Dipeptide	4.069	C_14_H_26_N_2_O_4_	309.1777	-	5.81	-
Benzoic acid	Phenolic acids	4.26	C_7_H_6_O_2_	-	181.0503	1.42	-
Tyrosyl-Leucine	Dipeptide	4.261	C_15_H_22_N_2_O_4_	295.1622	-	12.6	-
Hydroxyprolyl-Leucine	Dipeptide	4.423	C_11_H_20_N_2_O_4_	267.1321	-	-	3.70
2,6-dihydroxybenzoic acid	Phenolic acids	4.90	C_7_H_6_O_4_	-	153.0188	-	2.59
Resorcinol	Phenolic compound	4.91	C_6_H_6_O_2_	-	109.029	-	1.32
Myristicanol A	Phenylpropanoid	5.03	C_23_H_30_O_8_	-	433.182	1.89	-
2-N,6-N-Bis(2,3-dihydroxybenzoyl)-L-lysine	N-acylglycine	5.07	C_20_H_22_N_2_O_8_	-	477.1474	1.07	-
Lyoniresinol 9′-sulfate	Lignan	5.08	C_22_H_28_O_11_S	-	499.129	0.89	-
Tyrosyl-Valine	Dipeptide	5.08	C_14_H_20_N_2_O_4_	281.1478	-	-	3.72
Pantoyllactone glucoside	O-acyl carbohydrate	5.28	C_12_H_20_O_8_	-	351.1304	1.25	-
Kanzonol I	Isoflavonoid	5.29	C_27_H_32_O_5_	437.2321	-	2.69	-
Ganodermic acid Jb	Triterpenoid	5.30	C_30_H_46_O_4_	471.3495	-	1.95	-
Methyonyl-Methionine	Dipeptide	5.835	C_10_H_20_N_2_O_3_S_2_	-	339.1057	1.49	-
alpha-Hydrojuglone 4-O-b-D-glucoside	Glycoside	5.92	C_16_H_18_O_8_	-	337.092	-	1.98
Valyl-Tyrosine	Dipeptide	6.051	C_14_H_20_N_2_O_4_	303.1315	-	-	1.68
Vanillic acid	Phenolic acid	6.07	C_8_H_8_O_4_	-	167.0344	0.10	-
Quercetin 3-(2G-xylosylrutinoside)	Flavonoid glycosides	6.09	C_32_H_38_O_20_	-	741.1887	0.58	-
Kaempferol 3-sophoroside 7-glucoside	Flavonoid glycosides	6.11	C_33_H_40_O_21_	-	771.1999	-	2.61
3-(1,1-Dimethylallyl)scopoletin 7-glucoside	Coumarin	6.24	C_21_H_26_O_9_	-	467.1589	0.62	-
Quercetin 3-[rhamnosyl-(1->2)-rhamnosyl-(1->6)-glucoside]	Flavonoid glycosides	6.30	C_33_H_40_O_20_	-	755.2058	7.87	8.29
Isorhamnetin 3-O-[b-D-glucopyranosyl-(1->2)-[a-L-rhamnopyra-nosyl-(1->6)]-b-D-glucopyranoside]	Flavonoid glycosides	6.31	C_34_H_42_O_21_	-	785.2153	1.28	-
Quercetin 3,7-dirhamnoside	Flavonoid glycosides	6.47	C_27_H_30_O_15_	-	593,1522	3.04	5.01
Kaempferol 3-(2G-apiosylrobinobioside)	Flavonoid glycosides	6.53	C_32_H_38_O_19_	-	284.0314	4.37	-
Isorhamnetin 3-O-[b-D-glucopyranosyl-(1->2)-a-L-rhamnopyra-noside]	Flavonoid glycosides	6.77	C_28_H_32_O_16_	-	623.1618	1.53	-
3,6′-Disinapoyl sucrose	Phenylpropanoid glycoside	7.21	C_34_H_42_O_19_	-	799.2317	-	0.85
Quercetin 3-rhamnoside-7-glucoside	Flavonoid glycosides	7.27	C_27_H_30_O_16_	-	609.1476	-	6.05
Quercitrin	Flavonoid glycosides	7.35	C_21_H_20_O_11_	-	447.0942	-	0.77
Isovitexin 2″-O-[4-hydroxy-(E)-cinnamoyl-(->6)-beta-D-glucopyra-nosyl] 4′-O-beta-D-glucopyranoside	Flavonoid glycosides	7.71	C_42_H_46_O_22_	-	901.2419	0.68	-
Caffeic acid	Phenolic acid	8.17	C_9_H_8_O_4_	-	179.0343	0.16	-
Auriculoside	Flavan glycoside	8.34	C_22_H_26_O_10_	-	449.1483	0.58	-
Cichoriin	Coumarin	8.45	C_15_H_16_O_9_	-	339.0713	-	1
CMP-N-glycoloylneuraminate	Amino sugar	8.60	C_20_H_31_N_4_O_17_P	-	629.1271	4.40	1.23
CMP-N-acetylneuraminic acid	Amino sugar	8.81	C_20_H_31_N_4_O_16_P	-	659.1401	1.12	-
6-Demethoxycapillarisin	Coumarin	8.93	C_15_H_10_O_6_	-	285.0401	0.40	-
Genistein 8-C-glucoside	Flavonoid glycosides	9.14	C_21_H_20_O_10_	-	431.0986	0.71	-
m-Coumaric acid	Phenolic acid	10.08	C_9_H_8_O_3_	-	209.045	0.29	-
Frangulin B	Anthraquinone	10.63	C_20_H_18_O_9_	-	401.0879	-	1.41
Flazine	Alkaloids	10.92	C_17_H_12_N_2_O_4_	-	307.0722	-	1.4
Anantine	Imidazoles alkaloids	11.06	C_15_H_15_N_3_O	-	252.1136	0.64	-
Funtumine	Steroidal alkaloids	11.43	C_21_H_35_NO	318.2782	-	3.44	-
Corchorifatty acid F	Fatty Acyls	11.56	C_18_H_32_O_5_	-	327.2182	-	1.48
(-)-Ormosanine	Pentacyclic alkaloid	12.17	C_20_H_35_N_3_	318.2916	-	1.55	-
9,10-Dihydroxy-12,13-epoxyoctadecanoate	Hydroxy fatty acid	12.19	C_18_H_34_O_5_	-	329.2343	-	1.92
Eudesmin	Lignan	12.24	C_22_H_26_O_6_	409.1593	-	2.47	-
Embelin	Para-benzoquinone	12.54	C_17_H_26_O_4_	-	293.1771	-	2.52
Moschamine	Hydroxyindoles	12.58	C_20_H_20_N_2_O_4_	-	351.1349	0.68	-
Convallasaponin A	Steroidal glycoside	16.31	C_32_H_52_O_9_	581.362	-	2.29	-
5-Hydroxy-7-(4-hydroxyphenyl)-1-phenyl-3-heptanone	Diarylheptanoids	18.02	C_19_H_22_O_3_	-	297.1529	-	3.1
Sarcostin	Steroid	23.69	C_21_H_34_O_6_	-	381.2319	-	1.23

RT: Retention time.

**Table 7:** Binding energy values of docked compounds within in the LOX binding cavity (PDB: 3v99).

Compounds	Binding Energy (kcal/mol)
Retronecine (Z)	−5.3
2-(1,2,3,4-Tetrahydroxybutyl)-6-(2,3,4-trihydroxybutyl) pyrazine (Z)	−5.3
Malic acid (F) (Z)	−4.6
Quercetin 3-[rhamnosyl-(1->2)-rhamnosyl-(1->6)-glucoside] (F) (Z)	−9.2
Quercetin 3,7-dirhamnoside (F) (Z)	−8.7
Kaempferol 3-(2G-apiosylrobinobioside) (F)	−8.4
Quercetin 3-rhamnoside-7-glucoside (Z)	−8.0
CMP-N-glycoloylneuraminate (F)	−6.2
Prolyl-arginine (Z)	−6.9
Isoleucyl-Hydroxyproline (F)	−5.3
Valyl-Phenylalanine (F)	−7.2
N-Acetyl-leucyl-leucine (F)	−5.9
Tyrosyl-Leucine (F)	−6.2
Dexamethasone (R)	−8.2

(F): Compound identified in *Farsetia aegyptia* extract. (Z): Compound identified in *Zilla spinosa* extract. (R): Reference drug.

## Data Availability

All data generated or analyzed during this study are included in this published article.

## Supplementary Materials

The following supporting information can be downloaded at: https://www.mdpi.com/article/10.3390/plants15040523/s1, Table S1: Anti-inflammatory effect of *Zilla spinosa* and *Farsetia aegyptia* extracts on carrageenan-induced paw edema in rats; Table S2: Inhibitory effect (%) of *Zilla spinosa* and *Farsetia aegyptia* samples on xylene-induced ear edema in mice; Table S3: Analgesic activity of *Zilla spinosa* and *Farsetia aegyptia* extracts evaluated by the acetic acid-induced writhing test in mice; Table S4: Analgesic activity of *Zilla spinosa* and *Farsetia aegyptia* extracts evaluated by the hot plate test in mice at different time intervals.

## Abbreviations

The following abbreviations are used in this manuscript:

HR-LCMS	High-Resolution–Liquid Chromatography Mass Spectrometry
Faw	*Farsetia aegyptia* water
Zsw	*Zilla spinosa* water
%	percentage
mg/Kg	milligrams per kilogramme
IC_50_	Inhibition concentration
mg/mL	milligrams per milliliter
h	hour
Pdb	Protein data bank
Kcal/mol	Kilocalorie per mole
LOX	anti-lipoxygenase assays
LD_50_	Lethal doses
Zse	*Zilla spinosa* ethanol
Fae	*Farsetia aegyptia* ethanol
Zsc	*Zilla spinosa* chloroform
Fac	*Farsetia aegyptia* chloroform
BSA	Bovine Serum Albumin
min	minute
g/moL	Gram per mol
g	Gram
mL	milliliter
M	Molar
°C	Degree Celsius
nm	nanometer
µg/mL	Microgram per milliliter
UHPLC-PDA	Ultraviolet High-Phase Liquid Chromatography–Photo Diode Array
SPSS	Statistical Package for the Social Sciences
