# Supplementary material for: Chemical Profiling, Analgesic and Anti-Inflammatory Activities of Farsetia aegyptia and Zilla spinosa: Integrated In Vitro, In Vivo, and In Silico Studies
Source: Plants (Basel). 2026 Feb 7;15(4):523. doi: 10.3390/plants15040523 (PMC12944300; doi:10.3390/plants15040523)
Supplement: Supplementary file 1 [file plants-15-00523-s001.zip › plants-4061928-supplementary.pdf]

Article

Chemical Profiling, Analgesic and Anti-Inflammatory Activities of *Farsetia aegyptia* and *Zilla spinosa*: Integrated *In Vitro*, *In Vivo*, and *In Silico* Studies

Malek Besbes[1*], Assia Hamdi[2], Kaouther Majouli[3], Mabrouk Horchani[4], Abeer Ayed Alshammari[1], Saoussen Jilani[1], Salwa Ahmed Lotfi[1], Ramzi Hadj Lajimi[5], Hichem Ben Jannet[4], Walid Ben Selma[6], and Jamil Kraiem[2]

[1] Department of Biology, College of Sciences, University of Hail, P.O. Box 2440, Ha'il City 81451, Saudi Arabia; m.besbes@uoh.edu.sa (M.B); a.ashammar@uoh.edu.sa (A.A.A); s.jilani@uoh.edu.sa (S.J); sal.abdelmohsen@uoh.edu.sa (S.A.L)

[2] Laboratory of Chemical, Pharmaceutical and Pharmacological Development of Drugs, Faculty of Pharmacy, University of Monastir, Monastir 5000, Tunisia; assia.hamdi@fphm.u-monastir.tn (A.H); abderrahmene.bouraoui@fulbrightmail.org (A.B); (jamil.kraiem@fphm.u-monastir.tn) (J.K)

[3] College of Science and Humanities-Al-Duwadmi, Shaqra University, Riyadh, Saudi Arabia. kmajoli@su.edu.sa (K.M)

[4] Laboratory of Heterocyclic Chemistry, Natural Products and Reactivity (LR11Es39), Medicinal Chemistry and Natural Products, Faculty of Science of Monastir, University of Monastir, Avenue of Environment, Monastir 5000, Tunisia; (horchani.mabrouk@gmail.com) (M.H), (Hichem.BenJannet@fsm.rnu.tn) (H.B.J)

[5] Department of Chemistry, College of Science, University of Ha'il, P.O. Box 2440, Ha'il 81441, Saudi Arabia. r.lajimi@uoh.edu.sa (R.H.L)

[6] Laboratory of Analysis, Treatment and Valorization of Environmental Pollutants and Products, Faculty of Pharmacy, Monastir University, Monastir, Tunisia. walid.bensalma@issatmh.u-monastir.tn (W.B)

* Correspondence: *Malek Besbes (m.besbes@uoh.edu.sa)

Table S1. Anti-inflammatory effect of *Zilla spinosa* and *Farsetia aegyptia* extracts on carrageenan-induced paw edema in rats

Samples	Paw edema (%)					
	Dose mg/Kg	1h	2h	3h	4h	5h
Vehicle (mm)		3.32±0.09	4.09±0.14	4.65±0.04	3.75±0.16	3.43±0.51
Fac	50	27.29±1.72f	37.58±1.38c	54.82±2.66de	64.46±4.29ef	70.81±1.67e
	25	22.71±3.48e	32.75±2.18b	50.85±1.09d	61.60±0.83e	62.12±0.80d
	12.5	20.63±1.56e	31.33±0.76b	43.49±0.22bc	45.58±1.20b	44.14±3.34ab
Fae	50	41.88±0.54i	56.33±2.53g	67.33±3.61g	63.35±2.90e	72.22±4.39ef
	25	14.79±1.57d	31.25±132b	47.46±1.89c	53.31±1.73c	62.42±3.78d
	12.5	4.69±0.31a	30.83±0.52b	39.88±1.55b	42.27±2.91ab	45.96±2.75b
Faw	50	37.71±2.90h	50.42±0.95f	71.16±3.88h	73.57±1.88g	79.49±1.22f
	25	36.56±2.48h	45.83±0.52de	61.22±0.71f	63.17±2.82e	70.91±2.92e
	12.5	31.77±1.48fg	41.42±1.70d	62.47±0.66f	63.44±3.61e	70.91±0.80e
	6.25	13.13±0.54d	31.33±2.75b	41.50±1.81b	45.21±3.75b	51.82±1.60d
	3.125	8.02±0.36c	19.67±0.38a	32.16±1.66a	38.12±0.73a	42.53±1.26a
Zsc	50	14.58±2.53d	35.08±0.88c	51.07±2.81d	59.85±3.20de	70.30±1.21e
	25	1438±0.54d	33.58±2.02b	44.15±1.81bc	45.12±2.73b	51.21±3.96d
	12.5	7.81±0.54bc	21.25±0.75a	31.05±2.05a	37.48±0.32a	40.00±1.99a
Zse	50	34.79±0.48gh	43.83±3.41d	66.89±6.96g	69.34±2.92g	80.61±0.61g
	25	30.00±1.36fg	42.67±0.14d	51.29±0.13d	57.64±2.30d	69.49±3.21e
	12.5	6.25±2.81b	32.08±2.25b	41.80±2.63b	44.38±2.95b	46.06±1.32c
Zsw	50	37.08±2.73h	52.25±2.46f	58.06±3.31e	63.44±1.57e	63.43±4.18d
	25	36.77±0.36h	52.42±0.72f	57.10±1.00e	61.05±3.83e	68.59±1.06e

	12.5	34.17±1.00gh	47.83±0.29e	55.26±0.46de	55.99±3.97d	65.05±0.17de
	6.25	27.71±0.65f	40.92±1.66d	41.06±3.21b	4797±0.42bc	48.99±0.97c
**Diclofenac**	25	40.30±3.70i	62.10±1.91h	68.91±2.86g	70.34±2.73g	74.76±6.42ef

Fac: *Farsetia aegyptia* chloroformic extract, **Fae:** *Farsetia aegyptia* ethanolic extract, **Faw:** *Farsetia aegyptia* water extract, **Zsc:** *Zilla spinosa* chloroformic extract, **Zse:** *Zilla spinosa* ethanolic extract, **Zsw:** *Zilla spinosa* water extract. Values are expressed as mean ± SD (n = 6). Diclofenac was used as a reference anti-inflammatory drug. The letters (a–i) indicate a significant difference among the extract doses according to the Duncan assay ($p < 0.05$).

Table S2. Inhibitory effect (%) of *Zilla spinosa* and *Farsetia aegyptia* samples on xylene-induced ear edema in mice

Dose mg/Kg	Samples						Control	Reference
	**Fac**	**Fae**	**Faw**	**Zsc**	**Zse**	**Zsw**	**Vehicle**	**Dex (5 mg/mL)**
							0.11 ±0.03	66.24±3.10
**50**	80.91±1.29a	77.95±3.95a	97.05±2.81c	82.73±5.80ab	80.00±4.07a	96.67±2.92c		
**25**	78.18±5.35b	64.73±4.23a	88.18±6.72c	75.27±6.41b	60.18±1.19a	94.55±3.80d		
**12.5**	57.50±4.09a	56.18±4.87a	83.64±7.61c	65.45±4.17b	56.36±4.07a	85.68±8.83c		
**6.25**	58.48±0.52d	36.36±4.33b	70.23±9.60e	44.24±1.05c	21.82±4.98a	77.88±3.44f		
**3.125**	36.06±0.52a		67.58±4.10c			62.50±7.62b		
**1.56**			58.41±4.22			56.14±4.66		
**0.78**			51.14±5.05			34.55±4.98		
**0.39**			32.42±3.78					

Fac: *Farsetia aegyptia* chloroformic extract, **Fae:** *Farsetia aegyptia e*thanolic extract, **Faw:** *Farsetia aegyptia* water extract, **Zsc:** *Zilla spinosa* chloroformic extract, **Zse:** *Zilla spinosa* ethanolic extract, **Zsw:** *Zilla spinosa* water extract, Dex: dexamethasone. Values are expressed as mean ± SD (n = 6). The letters (a–f) show an important difference between the different doses of extracts in accordance with the Duncan assay ($p < 0.05$).

Table S3. Analgesic activity of *Zilla spinosa* and *Farsetia aegyptia* extracts evaluated by the acetic acid-induced writhing test in mice

	**% inhibition of writhing (acetic acid)**							
**Samples (mg/Kg)**	**50**	**25**	**12.5**	**6.25**	**3.125**	**1.562**	**0.78**	**0.39**
**Zsc**	77.27±3.79a	73.97±2.48bc	55.79±1.43a	36.36±2.58b				
**Zse**	79.75±0.72a	62.40±1.89a	57.85±1.24a	50.83±2.58c	25.62±1.24a			
**Zsw**	83.47±0.72b	78.93±1.24c	75.21±1.24c	72.31±0.72e	68.18±0.72d	53.31±0.72	42.15±1.89	
**Fac**	76.67±2.06a	70.39±2.33b	63.21±2.06b	31.81±2.06a				
**Fae**	78.02±2.06a	68.60±2.06b	60.07±1.55b	56.03±2.06d	40.78±2.69b			
**Faw**	81.61±0.78ab	80.71±0.78d	77.57±2.06c	75.33±0.78ef	64.56±1.55c	57.83±2.06	52.45±0.78	10.72±1.55
**ASL 200mg/Kg**	66.53±2.38							
**Vehicle (writhes)**	80.67±5.32							

Fac: *Farsetia aegyptia* chloroformic extract, **Fae:** *Farsetia aegyptia* ethanolic extract, **Faw:** *Farsetia aegyptia* water extract, **Zsc:** *Zilla spinosa* chloroformic extract, **Zse:** *Zilla spinosa* ethanolic extract, **Zsw:** *Zilla spinosa* water extract. **ASL:** acetylsalicylate of lysine. Values are expressed as mean ± SD (n = 6). Different letters (a–f) within the same column show an important difference between the different doses of extracts in accordance with the Duncan assay ($p < 0.05$).

Table S4. Analgesic activity of *Zilla spinosa* and *Farsetia aegyptia* extracts evaluated by the hot plate test in mice at different time interval

		Times (min)			
**Samples**	**Dose (mg/Kg)**	**30**	**60**	**90**	**120**
**Vehicle**		8.33±0.58a	9.67±0.58a	7.67±0.58a	5.33±0.58ab
**ASL**	200	25.33±1.53d	23.33±1.53d	20.00±2.00d	16.33±0.58cd
**Zsc**	50	23.00±1.00c	20.00±1.00d	14.67±0.58c	9.33±0.58b
	25	18.00±1.00bc	12.33±0.58b	10.00±1.00b	5.33±0.58ab
**Zse**	50	23.00±1.00c	1.00±1.00c	15.67±0.58c	8.33±0.58b
	25	18.33±0.58bc	17.67±0.58c	10.67±0.58b	4.33±0.58a
**Zsw**	50	25.00±1.00d	23.33±0.58d	20.00±0.00d	18.33±0.58d
	25	23.67±0.58c	20.33±0.58d	18.33±0.58cd	13.67±1.15c
	12.5	17.67±0.58bc	14.33±0.58b	10.33±0.58b	7.67±0.58b
	6.25	15.00±1.00b	11.33±1.15b	8.67±0.58a	4.33±0.58a
**Fac**	50	22.67±0.58c	20.33±0.58d	13.33±0.58bc	8.67±0.58b
	25	20.33±0.58c	17.67±0.58c	13.33±0.58bc	6.33±0.58ab
	12.5	14.00±1.00b	8.33±0.58a	5.33±0.58a	3.00±0.00a
**Fae**	50	23.33±0.58c	18.33±0.58c	15.00±1.00c	8.67±1.15b
	25	15.33±0.58b	10.33±0.58ab	7.67±0.58a	3.67±0.58a
**Faw**	50	25.67±0.58d	23.33±1.15d	19.67±0.58d	18.67±0.58d
	25	24.33±0.58cd	21.67±0.58d	19.00±1.00d	13.00±0.00c
	12.5	18.67±0.58bc	14.33±0.58b	10.67±0.58b	7.00±1.00b
	6.25	14.33±0.58b	8.00±1.00a	5.67±0.58a	3.33±0.58a

Fac: *Farsetia aegyptia* chloroformic extract, **Fae:** *Farsetia aegyptia* ethanolic extract, **Faw:** *Farsetia aegyptia* wáter extract, **Zsc:** *Zilla spinosa* chloroformic extract, **Zse:** *Zilla spinosa* ethanolic extract, **Zsw:** *Zilla spinosa* water extract. **ASL:** acetylsalicylate of lysine, Values are expressed as mean ± SD (n = 6). Different letters (a–d) within the same column show an important difference between the different doses of extracts in accordance with the Duncan assay ($p < 0.05$)
